# Hepatitis B virus is degraded by autophagosome-lysosome fusion mediated by Rab7 and related components

**DOI:** 10.1007/s13238-018-0555-2

**Published:** 2018-06-06

**Authors:** Yong Lin, Chunchen Wu, Xueyu Wang, Thekla Kemper, Anthony Squire, Matthias Gunzer, Jiming Zhang, Xinwen Chen, Mengji Lu

**Affiliations:** 1 Institute of Virology, University Hospital Essen, University of Duisburg-Essen, 45117 Essen, Germany; 2 State Key Laboratory of Virology, Wuhan Institute of Virology, Chinese Academy of Science, Wuhan, 430071 China; 3 Institute for Experimental Immunology and Imaging, University Hospital Essen, University Duisburg-Essen, 45117 Essen, Germany; 4 Department of Infectious Diseases, Huashan Hospital, Fudan University, Shanghai, 200040 China

**Dear Editor,**

With an estimated 240 million chronically infected people worldwide, hepatitis B virus (HBV) infection is a major public health problem (Schweitzer et al., 2015). Despite more than 30 years of intense research, many aspects of the HBV life cycle still remain unknown.

Recent progress demonstrated that efficient HBV replication is dependent on autophagy (Liu et al., 2014; Xie et al., 2016; Lin et al., 2017). Autophagy is a conserved catabolic process by which long-lived proteins and damaged organelles are sequestered in the cytoplasm and removed for recycling and important for maintaining cellular homeostasis (Mizushima and Komatsu, 2011), and involves the formation of autophagosomes, known as early autophagy, and their fusion with lysosomes and lysosomal cargo degradation, known as late autophagy. HBV induces partial autophagy to facilitate its own replication. Several reports indicated that HBV induces partial autophagy to facilitate its own replication through the actions of hepatitis B x (HBx) protein and the small HBV surface protein (HBsAg) (Sir et al., 2010; Li et al., 2011; Liu et al., 2014). According to these findings, the HBV life cycle is closely bound to autophagy. Yet, the exact mechanism of how autophagic flux affects HBV replication remains unclear. Thus, we investigated how HBV gene expression, replication and assembly are associated with autophagy.

To examine the effect of different autophagic phases on HBV production, human hepatoma cells HepG2.2.15 (Fig. S1A) were treated with the PI3KC3 inhibitor 3-methyladenine (3-MA), the Rab7 inhibitor CID1067700 (CID) or the lysosome inhibitor chloroquine (CQ). Immunofluorescence microscopy showed that the number of LC3 puncta was decreased by 3-MA treatment and increased following treatment with CID and CQ. Moreover, the ratios of LC3II/Actin were applied for assessing the autophagic activity, with beta-actin used as the internal reference for normalization (Xie et al., 2016). Western blot analysis of cellular lysates revealed that 3-MA elevated the autophagic cargo p62 expression level but decreased the levels of LC3-II and hepatitis B core antigen (HBcAg) (Fig. S1B). However, inhibitors of late autophagy CID and CQ elevated the levels of p62 and HBcAg in HepG2.2.15 cells (Fig. S1B). Moreover, 3-MA reduced the amount of secreted and intracellular HBsAg, the levels of HBV DNA in culture supernatants and intracellular HBV RIs, while the CID and CQ significantly increased their production (Fig. S1C). The effect of different autophagy inhibitors was also confirmed in primary human hepatocytes (PHHs) (Fig. S1D and S1E). Consistently, blocking the initiation of autophagy reduced HBV replication but interference with its late phase resulted in increased HBV production.

As the autophagy is a process mediating the degradation of cargos within the autophagosomes, it should be assumed that a significant part of HBV proteins and other components may be eliminated by the autophagy. Rab7 belongs to a family of small GTPases and plays a central role in regulating endo-lysosomal membrane traffic (Wang et al., 2011; Inoue et al., 2015). Rab7 is also required for the maturation of late endosomes (LEs)/MVBs as well as autophagosomes by recruiting its effectors Pleckstrin homology domain containing protein family member 1 (PLEKHM1) and Rab7-interacting lysosomal protein (RILP), directing the trafficking of cargo along microtubules and participating in the fusion step with lysosomes (McEwan et al., 2015a). Moreover, Rab7 silencing prevents the fusion of autophagosomes and lysosomes (Liu et al., 2014).

Accordingly, we chose Rab7 as the target to modulate the cellular autophagic process and to determine its role in the HBV life cycle. As shown in Figure 1A and 1B, Rab7 expression is decreased in hepatoma cells in the presence of HBV. Using specific siRNA, Rab7 silencing increased the number of LC3 puncta (Figs. 1C and S2A) and the levels of the autophagic cargo, LC3-II and p62, strongly increased after Rab7 silencing (Fig. 1D). Rab7 silencing significantly increased the amounts of HBcAg, HBV capsid and capsid-associated HBV DNA (Fig. 1D), and the levels of secreted HBsAg in culture supernatants and intracellular HBsAg, and intracellular HBV DNA in Huh7 and HepG2.2.15 cells (Figs. 1E and S2B), but not HBeAg production. These data along with results of Rab7 inhibition by CID (Fig. S1C) suggest that Rab7 silencing promotes HBV production by inducing incomplete autophagy.

**Figure 1:**
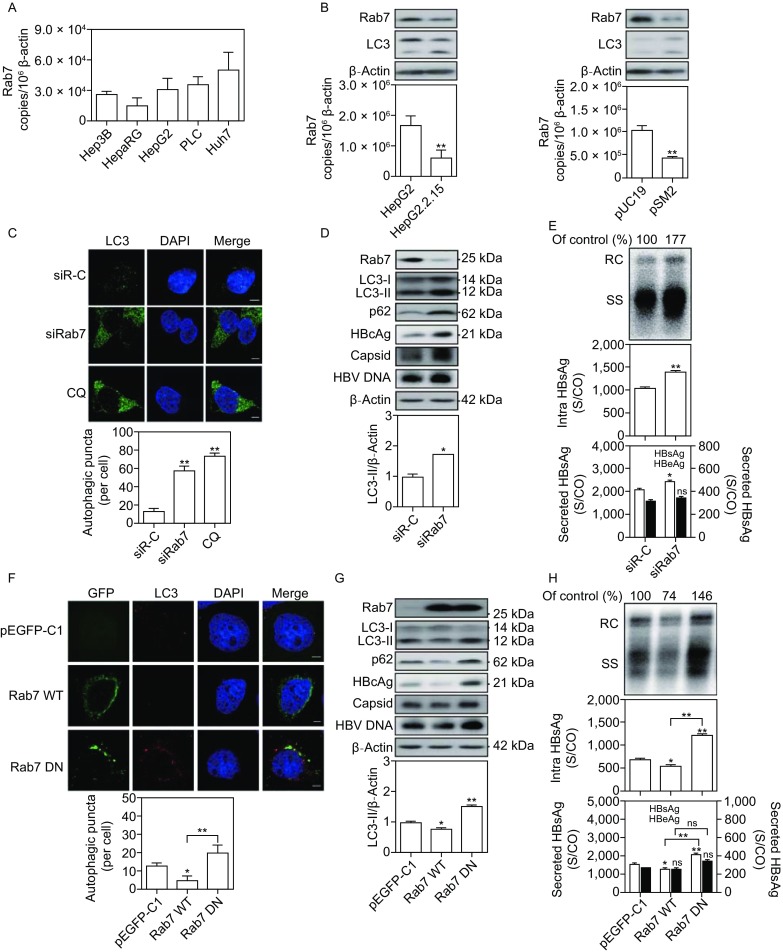
**Rab7 silencing promotes HBV replication and HBsAg production, but Rab7 activation decreases**. (A) The mRNA levels of Rab7 in different hepatoma cell lines, including Hep3B, HepaRG, HepG2, PLC and Huh7 cells, were determined by real-time (RT)-PCR using specific primers. (B) The protein and mRNA levels of Rab7 in HepG2 and HepG2.2.15 cells as well as in Huh7 cells after transfection with HBV plasmid pSM2 or control vector pUC19 were determined by Western blot and real-time RT-PCR, respectively. (C and D) HepG2.2.15 cells were transfected with 20 nmol/L siRNAs against Rab7 (siRab7) or control siRNA (siR-C). After 48 h, the transfected cells were fixed, incubated with primary antibody anti-LC3 and stained with Alexa Fluor 594-conjugated anti-rabbit secondary antibody IgG. The transfected cells were imaged by confocal microscopy. The cells were treated with 10 µmol/L CQ for 24 h as a positive control. Rab7, LC3, p62 and HBcAg expression and viral nucleocapsid levels were analyzed by Western blot. Detection of encapsidated HBV DNA was performed by Southern blot. (E) Huh7 cells were cotransfected with the pSM2 plasmid and siRab7 or siR-C at 20 nmol/L and harvested after 72 h. Analysis of secreted HBsAg and HBeAg in culture supernatants and intracellular HBsAg from cell lysates was performed by a chemiluminescent microparticle immunoassay (CMIA). Analyses of HBV replicative intermediates inside the cells were performed by Southern blot. (F) Huh7 cells were transfected with an expression vector carrying wild-type Rab7 (Rab7 WT), dominant negative Rab7 (Rab7 DN), or a control vector pEGFP-C1. After 48 h, the cells were imaged using a confocal microscopy. (G and H) Huh7 cells were cotransfected with pSM2 plasmid and Rab7 WT, Rab7 DN, or vector pEGFP-C1, and harvested after 72 h. S/CO: signal to cutoff ratio; RC: relaxed circular DNA; SS: single-stranded DNA. The data are shown as mean ± SEM. *P < 0.05; **P < 0.01; ns, not significant

Next, the effects of Rab7 activation on autophagy and HBV replication were examined in Huh7 cells by transfection of plasmids expressing wild-type and dominant negative Rab7 with HBV plasmid pSM2. The number of LC3 puncta was slightly decreased by overexpression of wild-type Rab7 (Fig. 1F) but increased following the expression of dominant negative Rab7. Rab7 overexpression also markedly degraded the autophagic cargo LC3-II and p62 (Fig. 1G) and significantly reduced the amount of HBcAg, HBV capsid and capsid-associated HBV DNA, as well as the levels of secreted HBsAg in culture supernatants and intracellular HBsAg, and intracellular HBV DNA (Fig. 1H). These results consistently demonstrate that Rab7 activation affects the autophagic degradative process and decreases HBV production. In contrast, the expression of dominant negative Rab7 clearly showed the opposite effect to the wild-type counterpart (Fig. 1G and 1H). The findings here also strongly suggest that autophagic degradation after autophagosome-lysosome fusion may be a major cellular pathway for reducing HBV loads in cells.

As the biological functions of Rabs depend on the interaction with their downstream effectors, we addressed whether Rab7 effectors play a critical role in the regulation of HBV production. So far, PLEKHM1 has been characterized as a specific effector for the terminal fusion of autophagosome and lysosomes. Thus, we assessed the effect of silencing PLEKHM1 on the autophagy flux by the detection of LC3 expression in HepG2.2.15 and Huh7 cells. Consistently, PLEKHM1 silencing increased the number of LC3 puncta (Figs. 2A and S3A). Western blot analysis showed that the levels of LC3-II and p62 were elevated by PLEKHM1 silencing (Fig. 2B), indicating a blockage of autophagic degradation. Next, the effects of PLEKHM1 silencing on HBV production were examined. PLEKHM1 silencing significantly increased the amounts of HBcAg (Fig. 2B), the levels of secreted HBsAg in culture supernatants, and intracellular HBsAg and HBV DNA, but not HBeAg production (Figs. 2C and S3B). Thus, PLEKHM1 participates in regulating the autophagic degradative process and HBV production.

**Figure 2:**
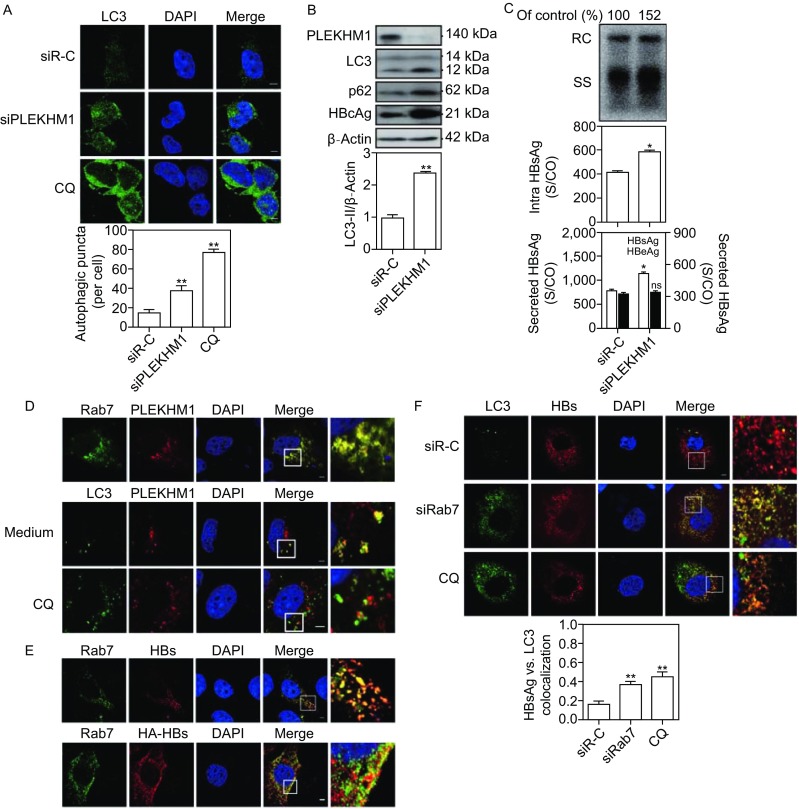

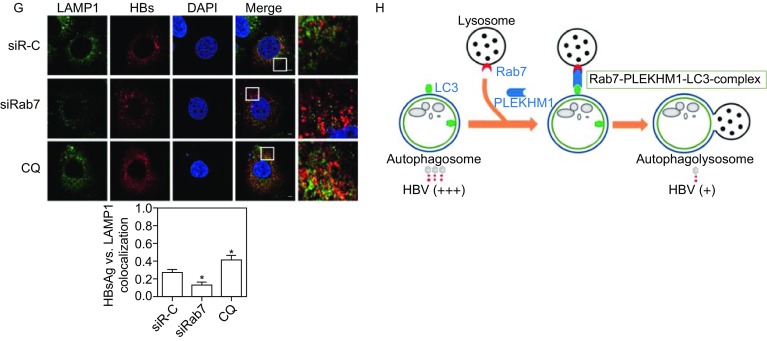
**Rab7 silencing interferes with the autophagic degradation of HBV by blocking the autophagosome-lysosome fusion**. (A and B) HepG2.2.15 cells were transfected with 20 nmol/L siRNAs against PLEKHM1 (siPLEKHM1) or control siRNA (siR-C). After 48 h, the transfected cells were imaged by confocal microscopy. PLEKHM1, LC3, p62 and HBcAg expression were analyzed by Western blot. (C) Huh7 cells were cotransfected with the pSM2 plasmid and siRab7 or siR-C at 20 nmol/L and harvested after 72 h. Analysis of secreted HBsAg and HBeAg in culture supernatants and intracellular HBsAg from cell lysates was performed by a chemiluminescent microparticle immunoassay (CMIA). Analyses of HBV genomes in culture supernatants and HBV replicative intermediates inside the cells were separately performed as described above. (D) Huh7 cells were cotransfected with plasmids GFP-Rab7 and DesRed-PLEKHM1, or GFP-LC3 and DesRed-PLEKHM1, and harvested after 48 h. The colocalization of LC3, Rab7 and PLEKHM1 was determined by confocal microscopy. (E) Huh7 cells were firstly transfected with mCherry-HBs. After 48 h, the cells were incubated with primary antibody rabbit anti-Rab7 and then stained with Alexa Fluor 488-conjugated anti-rabbit secondary antibody IgG (upper panel). Huh7 cells were cotransfected with HA-HBs, followed by incubating with primary antibody mouse anti-HA and rabbit anti-Rab7 and then staining with Alexa Fluor 488-conjugated anti-rabbit secondary antibody IgG and Alexa Fluor 594-conjugated anti-mouse secondary antibody IgG (bottom panel). The colocalization of Rab7 and HBsAg was determined by confocal microscopy. (F and G) Huh7 cells were transfected with mCherry-HBs and harvested after 48 h. Next, the cells were fixed, incubated with primary antibody rabbit anti-LC3 or anti-LAMP1, followed by staining with Alexa Fluor 488-conjugated anti-rabbit secondary antibody IgG. Cells cultured with 10 µmol/L CQ for 48 h were used as a positive control. The colocalization of HBsAg and LC3 or LAMP1 was determined by confocal microscopy. (H) A proposed model depicting that the autophagic degradation of HBV is regulated by Rab7-PLEKHM1-LC3 complex. A part of HBsAg and HBV virions may be degraded following the fusion of autophagosomes and lysosomes. Rab7 has different roles in the transport of autophagosomes and late endosomes (LEs)/MVBs, particularly controlling the fusion process of autophagosomes with lysosomes. Silencing Rab7 and its related components led to accumulation of autophagosomes/MVBs and an increase in intracellular and released HBsAg and HBV virions due to decreased fusion to lysosomes. S/CO: signal to cutoff ratio; RC: relaxed circular DNA; SS: single-stranded DNA. The data are shown as mean ± SEM. *P < 0.05; **P < 0.01; ns, not significant

Previous findings showed that PLEKHM1 contains some functional domains that could directly bind to LC3/GABARAP, Rab7 and HOPS complex (McEwan et al., 2015a; McEwan et al., 2015b), thus, may simultaneously bind to LC3 and Rab7. The interactions of Rab7, PLEKHM1 and LC3 were demonstrated in Huh7 cells by confocal microscopy. Partial co-localization of Rab7, PLEKHM1 and LC3 was observed by co-transfection of GFP-Rab7 and DesRed-PLEKHM1, or GFP-LC3 and DesRed-PLEKHM1 (Fig. 2D). Likely, PLEKHM1 serves as a platform linking both Rab7 and LC3 and directly bridges autophagic and lysosomal membranes, thereby facilitating the fusion of these vesicles.

As HBV production may be associated with autophagosomes, the interaction of HBsAg and Rab7 was further examined by confocal microscopy. HBsAg was found to partially co-localize with Rab7 in Huh7 cells (Fig. 2E), suggesting that HBsAg production is associated with autophagic flux controlled by Rab7. Rab7 silencing and CQ treatment led to increase of the colocalization of HBsAg with LC3 (Fig. 2F). Blocking autophagosome-lysosome fusion by Rab7 silencing or lysosomal functions by CQ treatment may prevent the autophagic degradation of intracellular HBsAg. Confocal microscopic analysis confirmed that the colocalization coefficient of LAMP1, a marker of lysosomes, with HBsAg was markedly decreased by Rab7 silencing but markedly elevated by CQ treatment (Fig. 2G), supporting the idea of the lysosomal degradation of HBsAg in the normal autophagic process. Blocking the autophagic degradation of HBsAg may result in an accumulation of HBsAg in these compartments. As Rab7-PLEKHM1-LC3 complex plays a critical role in autophagosome-lysosome fusion, we assume that Rab7-PLEKHM1-LC3 complex can regulate HBV autophagic degradation to some extent (Fig. 2H).

Finally, the effects of Rab7 silencing on HBV transcription and promoter activity were determined. However, Rab7 silencing did not change HBV RNA levels (Fig. S4A), suggesting that increased HBsAg production after Rab7 silencing is not due to transcriptional regulation. Moreover, there was also no effect on HBV promoter activity by silencing Rab7 (Fig. S4B).

Taken together, we examined how HBV replication was modulated by late phase of autophagy in the present study. While autophagy initiation was important for efficient HBV replication, a complete autophagic process led to the degradation of a significant part of HBV virions and HBsAg (Xie et al., 2016; Lin et al., 2017). Thus, the autophagic process may play a central role in HBV replication and assembly. In contrast, the expression and export of another secretory viral protein HBeAg was obviously less or not dependent on autophagy. Consistent with our results, Inoue et al. reported that the intracellular and secreted of LHBs and HBV virions were significantly increased by siRNA-mediated depletion of Rab7 (Inoue et al., 2015). Unfortunately, they did not examine HBV replication and explore the underlying mechanisms mediated by Rab7. Interestingly, the phosphoprotein of human parainfluenza virus type 3 is necessary and sufficient to inhibit autophagosome degradation by binding to SNAP29 and inhibiting its interaction with STX17 (Ding et al., 2014). This prevents the two host SNARE proteins from mediating autophagosome-lysosome fusion and results in increased viral production. Thus, efficient viral replication may be dependent on partial autophagy but needs to avoid the complete degradation process. In this regard, HBx protein may play an important role in inhibiting autophagic degradation (Liu et al., 2014).

Previously, PI3KC3 inhibition by 3-MA and silencing of ULK1 and other relevant components for autophagy initiation consistently led to decreased HBV replication, HBsAg formation and virion production (Li et al., 2011; Lin et al., 2017). Our data showed that Rab7 silencing did not change the levels of HBV RNA, indicating that late autophagy decreased HBsAg production without inhibiting HBV transcription levels. HBV envelopment has been proposed to occur at post-ER/pre-Golgi membranes, where cytosolic nucleocapsids are packaged inside a lipid envelope integrated with viral envelope proteins (Huovila et al., 1992). The autophagy pathway may additionally enhance HBV replication or envelopment by sequestering the necessary restriction factor(s) (Liu et al., 2014; Xie et al., 2016). Possibly, autophagosomes also provide a physical scaffold for HBV replication or envelopment (Sir et al., 2010; Lin et al., 2017). Finally, autophagosome membranes may be a source of membranes for viral envelopment (Patient et al., 2009; Li et al., 2011). These possibilities need to be tested in future studies.

## Footnotes

We thank Dr. Ruth Broering from the University Hospital Essen for providing primary human hepatocytes. This work was supported by grants from the Deutsche Forschungsgemeinschaft (RTG1949/1 and Transregio TRR60).

Yong Lin, Chunchen Wu, Xueyu Wang, Thekla Kemper, Anthony Squire, Matthias Gunzer, Jiming Zhang, Xinwen Chen and Mengji Lu declare that they have no conflict of interest. This article does not contain any studies with human or animal subjects performed by the any of the authors.

## Electronic supplementary material

Below is the link to the electronic supplementary material.

Supplementary material 1 (PDF 573 kb)
