# Supplementary material for: Hepatitis B virus is degraded by autophagosome-lysosome fusion mediated by Rab7 and related components
Source: Protein Cell. 2018 Jun 6;10(1):60–6. doi: 10.1007/s13238-018-0555-2 (PMC6321816; doi:10.1007/s13238-018-0555-2)
Supplement: Supplementary file 1 — Supplementary material 1 (PDF 573 kb) [file 13238_2018_555_MOESM1_ESM.pdf]

Supplementary Materials

Materials and Methods

Cell culture and transfection. The human hepatoma cell line HepG2.2.15 cells harboring integrated dimers of the HBV genome and Huh7 cells without an integrated HBV genome were used as described previously (Ni et al., 2010). Primary human hepatocytes were kindly provided by Dr. Ruth Broering (University Hospital Essen, Germany).

Plasmids and reagents. The plasmids pSM2 and GFP-LC3 have been described previously (Lin et al., 2016). The plasmids HA-HBs and mCherry-HBs are kept and stored in our lab. The plasmids GFP-Rab7 WT (12605), GFP-Rab7 DN (12660), and DsRed-PLEKHM1 (73592), were purchased from Addgene (Cambridge, MA, USA). The siRNA sequences used are shown in Table S1. Chemical inhibitors CID1067700 (SML0545), rapamycin (R8781), 3-MA (M928), and CQ (C6628) were purchased from Sigma-Aldrich (St. Louis, MO, USA).

Construction of the mCherry-HBs plasmid. The mCherry-HBs plasmid was constructed by inserting the HBsAg WT (nt160-841, GenBank accession number, AF282918), tissue plasminogen activator (tPA-SP), and mCherry tag sequences into a pcDNA3.1(+) backbone vector (Fan et al., 2008; Wu et al., 2012). A 69-bp signal sequence for tPA-SP has been used as a heterologous leader sequence (Jalah et al., 2007) to drive HBsAg into the cellular secretion pathway. The primers used for the construction of mCherry-HBs are listed in Table S2.

Analysis of HBV replication and gene expression. HBV RIs from intracellular core

particles were extracted from hepatoma cell lines and detected by Southern blot according to published protocols (Ni et al., 2010). HBV progeny DNA was extracted from culture supernatants using the DNA Blood Mini Kit (Qiagen, 51106) and quantified using quantitative real-time PCR (Invitrogen, 11733-046). HBV RNA in cells was measured using real-time RT-PCR assays (Qiagen, 204154) (the sequences of primers are shown in Table S2). The levels of intracellular HBsAg and secreted HBsAg and HBeAg in culture supernatants were determined using the Architect System and HBsAg and HBeAg CMIA kits (Abbott Laboratories, Chicago, IL, USA) according to the manufacturer's instructions. HBV nucleocapsids in the cell lysates were analyzed by native agarose gel electrophoresis and then detected by western blot. The encapsidated HBV DNA in nucleocapsids was detected by Southern blot.

Western blot analysis. Western blot analysis was performed as described previously (Lin et al., 2016). Briefly, prepared cells were washed with phosphate buffered saline and lysed with 1×lysis buffer (Cell Signaling, 7723). Protein samples were resolved by sodium dodecyl sulfate-polyacrylamide gel electrophoresis and then electro-transferred to nitrocellulose membranes. The membranes were incubated with the indicated primary antibodies overnight at 4 ℃ after being blocked with 5% milk in 1× TBST. Antibodies against the following proteins were used: anti-Rab7 (Cell Signaling, 9367), anti-PLEKHM1 (Cell Signaling, 66012), anti-Flag (Cell Signaling, 2368), anti-LAMP1 (Sigma, 9091), anti-HBcAg (Abcam, ab8637), anti-p62 (Abcam, ab91526), anti-LC3 (MBL, PM036), and beta-actin (Sigma, A5441). The membranes were washed with 1× TBST and incubated (as appropriate) with a secondary peroxidase-affiniPure Rabbit

anti-mouse IgG antibody (Jackson ImmunoResearch, 315-035-048) or a peroxidase-affiniPure goat anti-rabbit IgG antibody (Jackson ImmunoResearch, 111-035-045). Immunoreactive bands were visualized using an enhanced chemiluminescence system (GE Healthcare, RPN2106).

Real-time RT-PCR assay. Total RNA was extracted with TRIzol (Invitrogen, 15596-018), followed by digestion with the DNase Set (Roche, 10104159001). QuantiTect primer assays of human beta-actin (QT01680476) and Rab7 (QT00004949) were purchased from Qiagen Company. All the other sequences of primers used in the present study are shown in Table S2. For each sample, RT-PCR was performed in duplicate by using real-time RT-PCR assays (Qiagen, 204154). The expression levels of each gene are presented as values normalized against 10^6 copies of beta-actin transcripts.

Confocal microscopy. For immunofluorescence staining, Huh7 cells were grown on cover slips and co-transfected with the GFP-LC3 plasmid and siRNAs or other plasmids as indicated in each experiment. After 48 h, cells were washed with phosphate buffer solution, fixed in 4% paraformaldehyde, and permeabilized with 0.1% Triton X-100. The cells were incubated with primary antibodies and then stained with Alexa Fluor 488- (Jackson ImmunoResearch, 111-545-003), Alexa Fluor 594- (Jackson ImmunoResearch, 111-605-003), or Alexa Fluor 647- (Jackson ImmunoResearch, 111-605-003) conjugated Goat anti-Rabbit IgG (H+L). The nuclei were stained with 6-diamidino-2-phenylindole (DAPI), and the distribution of GFP-tagged LC3 protein was visualized with an LSM 710 confocal microscope (Zeiss, Jena, Germany) with a Plan-Apochromat 63×/1.40 oil Iris M27 objective. The number of LC3 puncta in cells was quantified as described previously (Lin et al., 2016). The co-localization of HBsAg and

relative organelle marker proteins was analyzed using ImageJ software.

Statistical analyses. Statistical analyses were performed using Graph Pad Prism software version 5.1 (La Jolla, CA, USA). Analysis of variance with two-tailed Student's *t* test or by one-way ANOVA with a Tukey posttest was used to determine significant differences. Differences were considered statistically significant when $P < 0.05$. All experiments were repeated independently at least three times.

Supplementary Figures

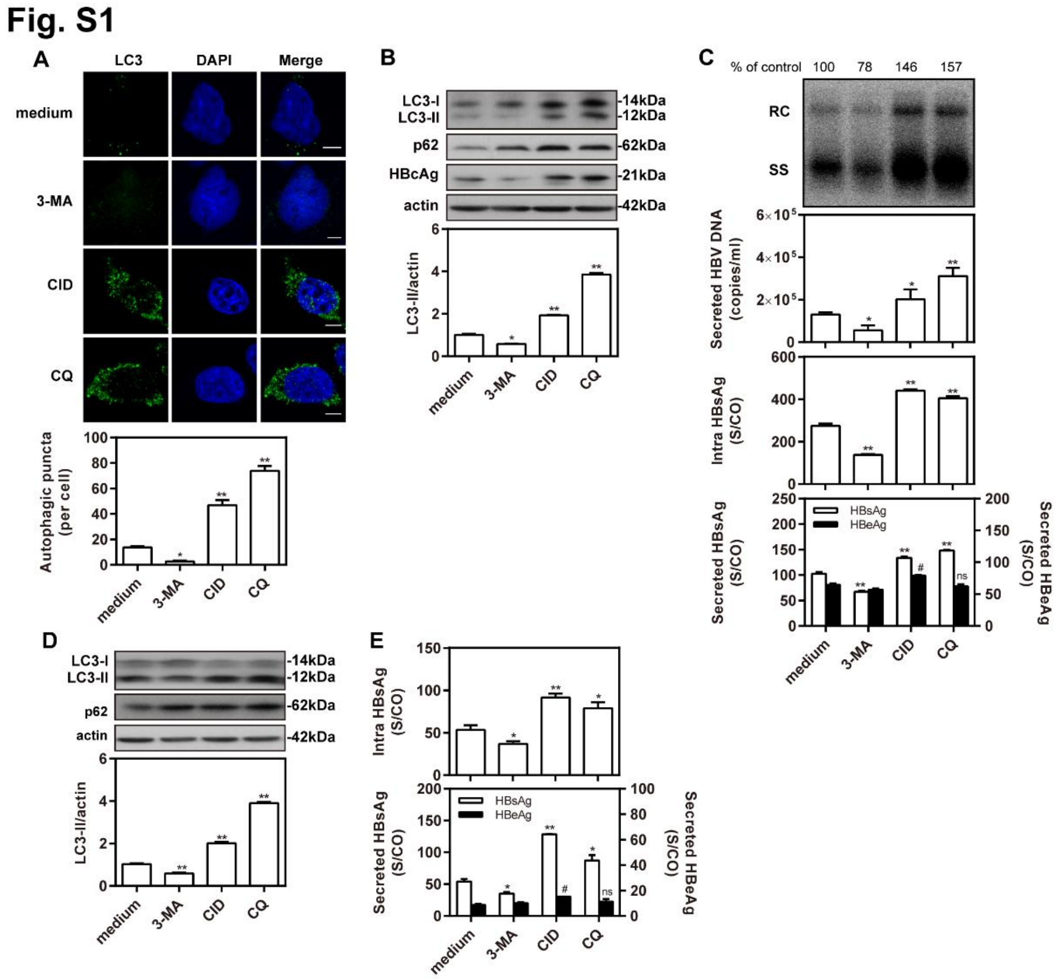

Figure S1. Inhibition of different autophagic phases inversely affects HBV replication. (A) HepG2.2.15 cells were treated with autophagy inhibitors 10 mM 3-methyladenine (3-MA), 5 μM CID1067700 (CID), and 10 μM chloroquine (CQ) for 48 h. LC3 puncta in the cells were analyzed by confocal microscopy. Bars, 5 μm. HepG2.2.15 cells (B and C) or primary hepatocytes with HBV virion infection at an MOI of 30 (D and E) were treated with autophagy inhibitors as in (A). LC3, p62, and HBcAg expression was analyzed by western blot with beta-actin as a loading control. Analysis of secreted HBsAg and HBeAg from culture supernatants and intracellular HBsAg from cell lysates was performed using a chemiluminescent microparticle

immunoassay (CMIA). The levels of HBV genomes in culture supernatants were determined by quantitative real-time PCR. HBV replicative intermediates in cells were detected by Southern blot. S/CO = signal to cutoff ratio; RC: relaxed circular DNA; SS = single-stranded DNA. The data are shown as mean ± SEM. *,# $P < 0.05$; **,## $P < 0.01$; ns, not significant.

Fig. S2

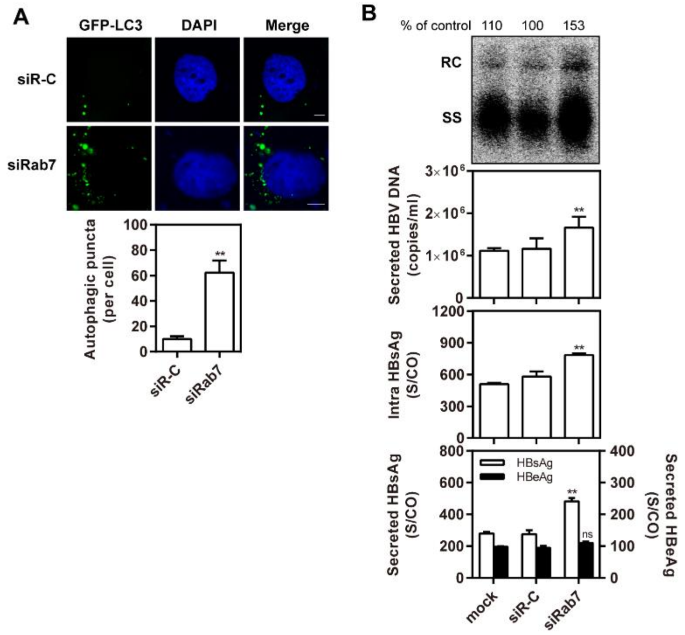

Figure S2. Rab7 silencing induces incomplete autophagy. (A) Huh7 cells were cotransfected with GFP-LC3 plasmids and 20 nM siRab7 or control siRNA (siR-C). After 48 h, the transfected cells were imaged by confocal microscopy. (B) HepG2.2.15 cells were transfected with 20 nM siRab7 or siR-C and harvested after 96 h. Analysis of secreted HBsAg and HBeAg in culture supernatants and intracellular HBsAg from cell lysates was performed by CMIA. Analyses of HBV genomes in culture supernatants and HBV replicative intermediates inside the cells were separately performed as described above. S/CO = signal to cutoff ratio; RC: relaxed circular DNA; SS = single-stranded DNA. The data are shown as mean ± SEM. * $P < 0.05$; ** $P < 0.01$.

Fig. S3

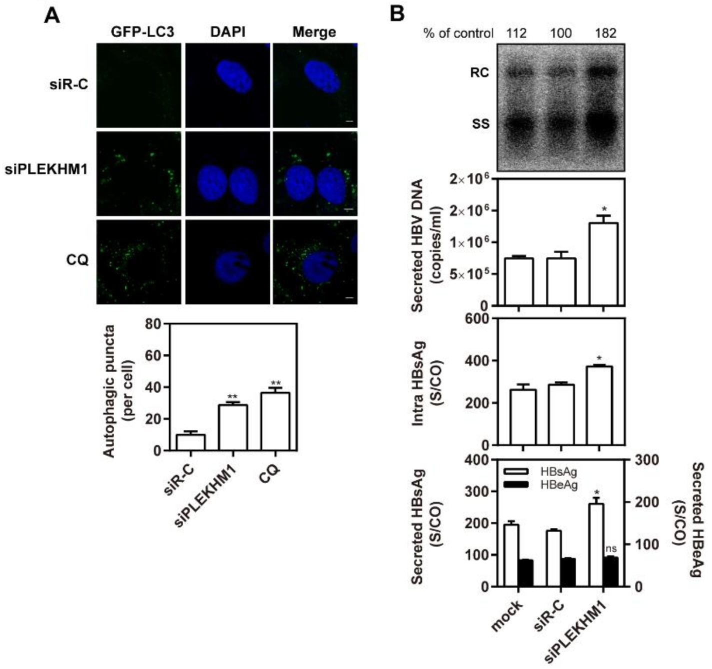

Figure S3. Silencing of Rab7 effector PLEKHM1 increases HBV replication and HBsAg production. (A) Huh7 cells were cotransfected with GFP-LC3 plasmids and 20 nM siRNAs against PLEKHM1 (siPLEKHM1) or control siRNA (siR-C). After 48 h, the transfected cells were imaged by confocal microscopy. The cells were treated with 10 µM CQ for 24 h as a positive control. (B) HepG2.2.15 cells were transfected with 20 nM siPLEKHM1 or siR-C and harvested after 96 h. Analysis of secreted HBsAg and HBeAg in culture supernatants and intracellular HBsAg from cell lysates was performed by CMIA. Analyses of HBV genomes in culture supernatants and HBV replicative intermediates inside the cells were separately performed as described above. S/CO = signal to cutoff ratio; RC: relaxed circular DNA; SS = single-stranded DNA. The data are shown as mean ± SEM. * $P < 0.05$; ** $P < 0.01$.

Fig. S4

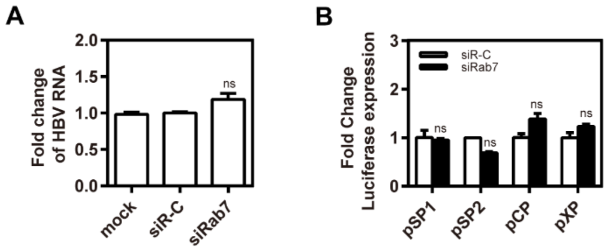

Figure S4. Rab7 silencing does not increase HBV transcription and promoter activity. (A) HepG2.2.15 cells were transfected with 20 nM siRab7 or control siRNA (siR-C) and harvested after 72 h. HBV RNA levels were analyzed by real-time RT-PCR assay. (B) Luciferase reporters containing the HBV promoter regions pSP1, pSP2, pCP, and pXP were co-transfected with siRab7 or siR-C at 20 nM into Huh7 cells, respectively. At 48 h, firefly and Renilla luciferase activities were analyzed using a Dual-Glo luciferase reporter assay. The relative luciferase expression was calculated by fold-change and normalized to siR-C. The data are shown as mean ± SEM. * $P < 0.05$; ** $P < 0.01$; ns, not significant.

Supplementary Tables

Table S1. List of siRNAs used in this study

Name	Product Name	Company	Target sequence
siR-C	Allstars Negative Control siRNA	Qiagen	Proprietary
siRab7	Hs_RAB7_5 FlexiTube siRNA	Qiagen	CACGTAGGCCTTCAACACAAT
siPLEKHM1	Hs_PLEKHM1_3 FlexiTube siRNA	Qiagen	CACCGCCATGGCCCACATTAA

Table S2 Primers used for Real-time PCR and cloning

Gene name	Application	Type	Sequence 5'-3'	Position of 5'-base
HBV DNA	real time PCR	Forward	GTTGCCCGTTTGTCCTCTAATTC	465
HBV DNA	real time PCR	Reverse	GGAGGGATACATAGAGGTTCCTT	563
HBV RNA	real time RT-PCR	Forward	CCGTCTGTGCCTTCTCATCT	1551
HBV RNA	real time RT-PCR	Reverse	TAATCTCCTCCCCCAACTCC	1756
F-tPA-SP	Cloning	Forward	GACAAGCTTATGGATGCAATGAAGAGAGGGCTCTGCTGTGTGCTGCTGCTGTGTG	Ref.(Fan et al., 2008; Wu et al., 2012)
R-tPA-SP	Cloning	Reverse	CCAGAATTCGCTGGGCGAAACGAAGACTGCTCCACACAGCAGCAGCACACAGCAGAG	Ref. (Fan et al., 2008; Wu et al., 2012)
F-mCherry	Cloning	Forward	CAGGAATTCATGGTGAGCAAGGGC	Ref. (Fan et al., 2008; Wu et al., 2012)
R-mCherry	Cloning	Reverse	GTGGGATCCTCCTGAACCCTTGTACAGCTCGTCCAT	Ref. (Fan et al., 2008; Wu et al., 2012)
F-HBsAg	Cloning	Forward	CGCGGATCCATGGAGAACATCACATCAGGA	155
R-HBsAg	Cloning	Reverse	CAGCTCGAGTTAAATGTATACCCAAAGACA	835

Genebank accession No. of the reference sequences: HBV real time PCR primer, V01460; HBsAg cloning, AF282918;

References

Fan, J.Y., Cui, Z.Q., Wei, H.P., Zhang, Z.P., Zhou, Y.F., Wang, Y.P., and Zhang, X.E. (2008). Split mCherry as a new red bimolecular fluorescence complementation system for visualizing protein-protein interactions in living cells. Biochem Biophys Res Commun 367, 47-53.

Jalah, R., Rosati, M., Kulkarni, V., Patel, V., Bergamaschi, C., Valentin, A., Zhang, G.M., Sidhu, M.K., Eldridge, J.H., Weiner, D.B., *et al.* (2007). Efficient systemic expression of bioactive IL-15 in mice upon delivery of optimized DNA expression plasmids. DNA Cell Biol 26, 827-840.

Lin, Y., Deng, W., Pang, J., Kemper, T., Hu, J., Yin, J., Zhang, J., and Lu, M. (2016). The microRNA-99 family modulates hepatitis B virus replication by promoting IGF-1R/PI3K/Akt/mTOR/ULK1 signaling-induced autophagy. Cell Microbiol 23, 546-545.

Ni, Y., Sonnabend, J., Seitz, S., and Urban, S. (2010). The pre-s2 domain of the hepatitis B virus is dispensable for infectivity but serves a spacer function for L-protein-connected virus assembly. J Virol 84, 3879-3888.

Wu, C.C., Deng, W.Y., Deng, L., Cao, L., Qin, B., Li, S.X., Wang, Y., Pei, R.J., Yang, D.L., Lu, M.J., *et al.* (2012). Amino Acid Substitutions at Positions 122 and 145 of Hepatitis B Virus Surface Antigen (HBsAg) Determine the Antigenicity and Immunogenicity of HBsAg and Influence In Vivo HBsAg Clearance. J Virol 86, 4658-4669.
